# Anthelmintic potency of *Rumex crispus* L. extracts against *Caenorhabditis elegans* and non-targeted identification of the bioactive compounds

**DOI:** 10.1016/j.sjbs.2021.09.026

**Published:** 2021-09-17

**Authors:** Oladayo Amed Idris, Olubunmi Abosede Wintola, Anthony Jide Afolayan

**Affiliations:** a Medicinal Plants and Economic Development (MPED) Research Centre, Department of Botany, University of Fort Hare, Alice 5700, South Africa; b Unit for Environmental Sciences and Management (UESM), Faculty of Natural and Agricultural Sciences, North-West University, Potchefstroom, North West, South Africa

**Keywords:** *Caenorhabditis elegans*, Gastrointestinal infections, Anthelmintic drug, Bioactive compounds, Phytochemical, Ethnoveterinary therapists

## Abstract

Traditional healers and ethnoveterinary therapists use several medicinal plants, such as *Rumex crispus* L., to treat endoparasite infections. *R. crispus* has been established by researchers to be effective agasint a few parasitic worms. In this study, we evaluated the potency of *R. crispus* extracts on the model organism, *Caenorhabditis elegans* and the bioactive compounds of the extracts were also identified. The solvent extracts of *R. crispus* were tested against *C. elegans* for up to 72 h. The effect of the extracts on *C. elegans* was examined using light microscopy (LM) and scanning electron microscopy (SEM). LM and SEM analysis showed damage on the body wall, reduced body and slight modifications of the nematode organs. The lethality test reveals a significant reduction in the viability of the nematode with the water extract of leaf (LF-WAE), among others, having the strongest potency against the nematode, with 83% lethality. Anlysis done with Fourier-transform infrared spectroscopy (FTIR) spectra reveals various characteristic vibration bands and fingerprint bands at 3400–600 cm^−1^, identifying phenols, organic acids, aromatics, amines, among others in the plant. The compounds were identified with liquid chromatography-mass spectrometry (LC-MS), under the categories of flavonoids, steroidal alkaloids and proanthocyanidin. In conclusion, this study confirmed that *R. crispus* has anthelmintic potential, using standardised *C. elegans* models as a tool and suggests that there could be novel compounds yet to be explored in the studied plant that could be of great benefit to livestock and humans.

## 1 Introduction

One of the major human global health challenges is parasitic worm infections. It brings about an increase in human and animal disease burden (Alum et al., 2010), leading to a considerable loss of manpower and livestock production globally. In the livestock industry, gastrointestinal (GI) infections caused by parasitic worms are among the main problems causing an increase in livestock mortality, reduced growth, a decrease in meat value and economic losses (Kumarasingha et al., 2014, Perry and Randolph, 1999). In an attempt to solve the problem of helminthic infections, several measures have been put in place to control the menace caused by the parasites, but the main methods adopted in developed countries are the use of anthelmintic compounds and improved sanitation (Kappagoda et al., 2011).

The infections caused by parasitic worms demand a new and effective anthelmintic approach, due to the failure of commercial drugs currently used in the control of helminthiases. Antihelmintic drugs have been reported to lose their efficacy over time due to resistance traits of parasites (Prichard, 2008, Sant’anna et al., 2013, Wolstenholme et al., 2004) and have side effects, particularly in children and pregnant women. The major classes of broad-spectrum anthelmintic drugs, Benzimidazoles, Macrocyclic Lactones, and Levamisole/Imidazothiazoles (Prichard, 2008, Sutherland and Leathwick, 2011), Piperazine and Paraherquamide (Idris et al., 2019a) and Amino-acetonitrile derivatives (AAD) (Aremu et al., 2012), have been reported to be resisted by side or major targeted parasitic worms (Idris et al., 2019a).

The various mechanisms of action of the drugs were once effective but declared futile after resistance. For instance, the case of resistance to ivermectin, morantel and oxfendazole was first reported in 1990 in New Zealand (Watson and Hosking, 1990). This calls for new drugs with different mechanisms of action. There have been a few holistic scientific examinations of traditional healers' claims about the use of medicinal plants for the treatment or prevention of diseases (Alves and Rosa, 2007). Plants, however, play an important role in therapy and their secondary metabolites are a reliable source for an alternative anthelminthic drug (Wink, 2012). Several medicinal plants have been documented (Jimoh et al., 2020, Jimoh et al., 2019). Some of which are reported to have anthelminthic potential (Wink, 2012). Folk-medicine and ethnoveterinary medicine use *Rumex crispus* to treat endoparasites and stomach related problems in humans and livestock (Lans et al., 2007). The decoction or infusion of the aerial parts and the root of *R. crispus* could also be used for the treatment of other health disorders; diarrhoea, constipation, inflammation, gallbladder disorders, piles, internal bleeding, wounds, and various skin problems (Idris et al., 2017, Vasas et al., 2015). Plant-derived anthelmintic compounds are promising for treating worms which are reported to be resistant to synthetic drugs (Shalaby, 2013). However, to compete effectively with synthetic drugs in the market, plant-based anthelmintic compounds should be degradable in the environment, have minimal side effects, have a lower toxic effect and leave no detrimental residues on animal products. On the contrary, the possibility of adverse effects of plant-derived compounds should not be underestimated. As such, screening for effective and nontoxic anthelmintic compounds in plants remains a major question in the development of novel drugs. The screening of helminthic infections *in vivo* or in the natural host is typically very expensive and can raise concerns about animal welfare, leading to ethical issues unlike research conducted on *Caenorhabditis elegans* or the use of other invertebrates. Consequently, the use of the nematode model, *Caenorhabditis elegans,* to test the efficacy of anthelmintic drugs is increasingly gaining attention because it is non-expensive and *C. elegans* is a free-living nematode (Desalermos et al., 2011, Sant’anna et al., 2013). The nematode has a short life cycle of 3.5 days at 20 °C (Muschiol et al., 2009), although the life cycle can be greatly influenced by food supply and environmental conditions in order to achieve fecundity. The worm has recently been used extensively in biomedical research (Kumarasingha et al., 2014) and high-throughput screening (HTS) (Swierczek et al., 2011), making *C. elegans* an ideal tool for screening anthelminthic drugs. A previous study revealed *C. elegans* shares genomic homology with *Strongyloides stercoralis* (Mitreva et al., 2004) and other parasitic worms. Although the complex life cycle of some parasitic worms makes it a challenge to examine the parasite in the transition stage, which can not be examined by using *C. elegans*, the free-living nematode offers a convenient model, instrumental in improving and studying the mechanism of action of many commercial anthelmintic compounds (Burns et al., 2015).

## 2 Materials and methods

In this study, *C. elegans* was used to examine the potency of eight extracts of *R. crispus* and levamisole was used as an anthelmintic reference. The micromorphology of the nematodes was examined using a scanning electron microscope (SEM) and an optical microscope. The compounds in the extracts of *R. crispus* were thereafter characterized by Fourier-transform infrared spectroscopy (FTIR) and liquid chromatography-mass spectrometry (LC-MS). The annotated spectrum of FTIR was used to interpret the characteristic functional groups in compounds based on the absorbance of light wavelength (Packialakshmi and Naziya, 2014) as used in this study. LC-MS, using positive ionization and the Mass-to-Charge Ratio (*m*/*z*), was used to isolate and identify compounds that contain heat-labile substituents or that are relatively polar.

### 2.1 Preparation of plant extracts

The plant was collected in two locations in the Eastern Cape, South Africa: Site 1 (32°47′30.2 “S, 26°50′51.1 ”E) and Site 2 (32°47′1.23 “S, 26°51′9.85 ”E). It was washed, the root was separated from the leaf, then air-dried until the weight was constant. The samples were then pulverized with an electric blender (Polymix PX-MFC90D Switzerland). The extraction was done with four solvents; methanol, ethanol, acetone and water (10:1 v/w), and then the filtrate of ethanol extract (ETE), acetone extract (ACE) and methanol extract (MEE) were concentrated with a rotary evaporator (Strike-202 Steroglass, Italy). The filtrate of water extract (WAE), on the other hand, was chilled at −40 °C and freeze-dried for 72 h.

The plant extracts were reconstituted with M9 buffer (6 g Na_2_HPO_4_, 3 g KH_2_PO_4_, 5 g NaCl, 0.25 g MgSO_4_·7H2O in 1 L of distilled water) as described (Sant’anna et al., 2013) and 1% Dimethyl sulfoxide (DMSO) was added to improve the dissolution of the stock. The mixture was vortexed until it completely dissolved and then sonicated for 60 min at 40 °C. The extracts were further filtered through 0.45 µm and then 0.22 µm sterile filters to clean and sterile the stock solution. Subsequently, the stock solutions (extracts) were prepared in concentrations of 0.125, 0.25, 0.5, 1.0, 2.0 and 5.0 mg extract/mL. The available commercial anthelmintic drug, levamisole, was prepared and used as the positive control.

### 2.2 Maintenance and isolation of adult *C. elegans*

The nematode (*C. elegans*); Bristol N2 wild-type strain, was obtained from the University of Pretoria, South Africa. It was grown on Nematode Growth Medium (NGM: 17 g agar, 2.5 g peptone from casein, 3 g NaCl, 0.5% cholesterol, 25 mM KH_2_PO4, 1 mM MgSO_4_, and 1 mM CaCl_2_ in 1 L of distilled water), seeded with *Escherichia coli* strain OP50, and incubated at 20 °C as described by Kumarasingha et al. (2014).

The nematodes in the cultured plates were washed with M9 solution to collect the adults and eggs. It was centrifuged at 800*g* for 5 min, the supernatant was discharged and the pellet was treated with a lysing solution (1% hypochloride and 5 M NaOH in distilled water) for 5 min. The pellet was then washed immediately, five times with M9 solution by centrifuge at 1000*g* for 10 min. The eggs were kept in M9 solution for 24 h at 20 °C to hatch to L1. The L1 larvae were further incubated in NGM at 20 °C for 48 h to allow them to grow into adults. The worms were collected, washed five times in M9 solution with a centrifuge at 450*g* for 3 min before being used for the assays. The research was conducted according to animal welfare and experimental guidelines. The study was approved by the University of Fort Hare, Animal Research Ethics Committee (UAREC) with certificate reference number: AFO121SIDR01.

### 2.3 Treatment of plant extracts on *C. elegans*

The assay was performed using 96 well microtitre plates with 250 µL of the graded media (extracts) in three replicates for each concentration. Negative and positive controls were 1% DMSO in M9 and 0.1 mg/mL levamisole in M9 respectively. Prior to the assay, the concentration of the extracts of *R. crispus* was assessed in the range of 0.5–5 mg/mL against *C. elegans*. The nematode was next examined for percentage lethality at 2 mg/mL *R. crispus*, a concentration that had a considerable impact on the nematode. This was in accordance with the dose used by Kumarasingha et al. (2014). A total of 30*C. elegans* were pipetted per well and the plates were sealed and incubated at 20 °C. The anthelmintic activity of the plant extracts on the adult nematode was examined at the interval of 12, 24, 36, 48 and 72 h exposure periods at 2 mg/mL of extracts and 0.1 mg/mL of levamisole. Nematodes are considered dead when the body is straight, there is no head, tail or pharyngeal movement for a period of 10 s of observation (Skantar et al., 2005). The lethal concentration (LC_50_) was thereafter extrapolated and viability (lethal endpoint) was assessed at 72 h.

### 2.4 Micromorphology analysis of treated *C. elegans* with *R. crispus* extracts

#### 2.4.1 Light microscopy (LM)

The micromorphology of the nematode was studied with a light microscope after being exposed to 2 mg/mL of the extracts and 0.1 mg/mL of levamisole. The examined nematodes were obtained from three independent experiments and 400 mM ethanol was added for 5 min to anaesthetize nematodes mounted on a glass slids. All samples collected were observed under an Olympus CX23 light microscope using differential interference contrast (DIC) and bright field. The micrograph was captured with a DinoCapture 2.0 digital camera.

#### 2.4.2 Scanning electron microscopy (SEM) analysis

An estimate of 90 nematodes was obtained from three independent experiments (2 mg/mL treatment) in each sample for SEM screening. Nematodes were fixed in 2.5% glutaraldehyde in 0.1 M cacodylate buffer, pH 7.4 at 4 °C for 24 h. It was rinsed three times in 0.1 M cacodylate buffer, pH 7.4 and centrifuged at 450*g* for 5 min. The pellets were post-fixed in a solution containing 2% osmium tetroxide and 1.25% potassium ferrocyanide, pH 7.4 for 24 h, followed by washing three times in 0.1 M cacodylate buffer. The pellets were then dehydrated in a graded series of ethanol solutions from 30 to 100% and samples were critical point-dried in liquid CO_2_ using Balzer's apparatus. All samples collected were observed with a JEOL JSM-6390LV Scanning Electron Microscope.

### 2.5 Non-targeted screening of chemical constituent of *R. crispus*

#### 2.5.1 Fourier transform infrared Spectrophotometer (FTIR)

The pulverized samples and the dried solvent extracts of *R. crispus* were analysed using JASCO FT-IR 4100 spectroscopy adopting the KBr disc technique. A translucent sample disc was created by encapsulating 100 mg of KBr pellet with 10 mg of the dried plant samples. Thereafter, it was loaded into FTIR spectroscopy and data of infrared transmittance was collected over the range of 500–4000 cm^−1^ with a resolution of 4 cm^−1^. Plain KBr pellets are blank and all samples were analysed in triplicates.

#### 2.5.2 Liquid chromatography–high-resolution mass spectrometry

Prior to the LC-MS analysis, 10 mg of each extract was dissolved in acetonitrile (ACN)/H_2_O (1:1 v/v) to obtain a final solution of concentration of 1 mg/mL. The chemical composition of the fractions of the extracts of *R. crispus* was determined using the HPLC-MS-UV technique. The detection and identification of the analytes were performed on an LC-MS-MS system. The chromatograph was equipped with an electrospray ionization (ESI) source for mass analysis and detection, then connected online to a 4000 QTRAP triple quadrupole tandem mass spectrometer (Applied Biosystems/MDS Sciex, Canada), for data collection and analysis. The separation column (150 × 4.60 mm and a particle size of 5 μm) was set at a temperature of 40 °C. The mobile phase was a mixture of H_2_O containing 0.1% formic acid (v/v) (A) and acetonitrile (B). The sample (5 μL) injected was set at a flow rate of 0.2 mL/min at the mobile phase with solvent A and B. The gradient elution system of the mobile phase was carried out using the following: 0 min, 2%B; 10 min, 15%B; 35 min 35%B; 46 min, 95%B and 55 min, 2%B. Ionization was performed in the positive electrospray mode and the turbo ion spray source was set as follows: capillary voltage: 3500 V, source temperature: 200 °C, collision energy: 10.0 eV, using Nitrogen as the collision and also nebulizing gas. The quantification was carried out in multiple reaction monitoring (MRM) mode.

#### 2.5.3 Statistical analysis

All data and statistical analyses were done with either Microsoft Excel, SPSS or Minitab depending on suitability. Data was compared using one-way ANOVA, Tukey test, at 95% confidence intervals (CI) and presented as mean ± standard error using Minitab. The LC_50_ (lethal concentration required to kill 50% of worms) values were obtained by using Probit analysis (SPSS version 21) followed by linear regression.

## 3 Results

### 3.1 Toxicological screening of plant extracts on *C. elegans*

Eight extracts obtained from the root and leaf of *R. crispus* were evaluated in this study and compared with levamisole (Positive control). The average extract yield of three independent extractions of the root are; RT-MEE (18.08 ± 1.73%), RT-ETE (7.28 ± 3.02%), RT-ACE (3.48 ± 0.15%) and RT-WAE (19.01 ± 2.90%), which were observed to be slightly higher than the yield of the leaf; LF-MEE (11.05 ± 3.52%), LF-ETE (5.21 ± 2.05%), LF-ACE (2.83 ± 0.11%), and LF-WAE (15.37 ± 3.00%). The extracts show a significant reduction in the viability of the adult *C. elegans* (Fig. 1a). In the first 24 h of the experiment, the lethality of extracts on the worms was quite moderate; RT-MEE (23%), RT-ETE (26.6%), RT-ACE (40%), LF-MEE (30%), LF-ETE (23.3%), LF-ACE (10%) and Levamisole (40%) except RT-WAE (53.3%) and LF-WAE (53.3%), whose results showed a rapid reduction in the nematode. In 72 h of treatment, the lethality of Levamisole was 100%. The nematode exposed to methanol extract of the root, methanol extract of the leaf, ethanol extract of the leaf and acetone extract of the leaf survived by more than 50%. The water extract of the leaf (83%) has the highest potency against the nematode, followed by water extract of the root (73.3%), acetone extract of the root (73.3%) and ethanol extract of the root (66.6%). It was also observed that extracts with high potency and levamisole induced the phenomenon called *endotokia matricida*, where eggs hatch internally in the adult worm, resulting in the rupture of adult nematodes due to internal larvae movement (Fig. 2, Fig. 3). The LC_50_ (mg/mL) and the confidence limits of the concentrations of extracts were recorded in Table 1. The LC_50_ of LF-WAE (0.11 mg/mL) was the lowest, indicating a strong potency against *C. elegans*, followed by the LC_50_ of RT-WAE (0.13 mg/mL) and RT-ACE (0.29 mg/mL) among others, which indicate these extracts are toxic to the nematode. As evaluated by the probit analysis in Table 1, the dose–response curve (Fig. 1b) illustrates the lethal dose (LD) of the most active treatments and the reference compound at 72 h.

**Fig. 1a:**
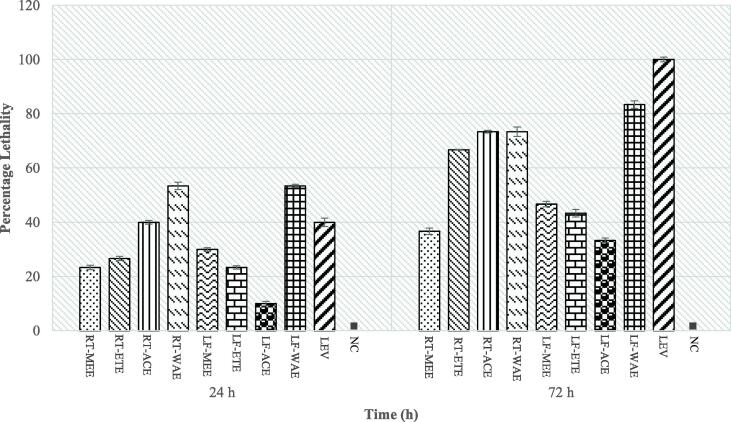
The effects of *R. crispus* extracts, at 2 mg/mL, on the Bristol N2 wild-type strain of *Caenorhabditis elegans* up to 72 h. Bars and error bars represent mean and standard errors. NC (■) denotes the negative control (M9 media with 1% DMSO); RT-MEE: methanol extract of the root; RT-ETE: ethanol extract of the root; RT-ACE: acetone extract of the root; RT-WAE: water extract of the root; LF-MEE; methanol extract of the leaf; LF-ETE: ethanol extract of the leaf; LF-ACE: acetone extract of the leaf; LF-WAE: water extract of the leaf; LEV: Levamisole (0.1 mg/mL), the positive control.

**Fig. 1b:**
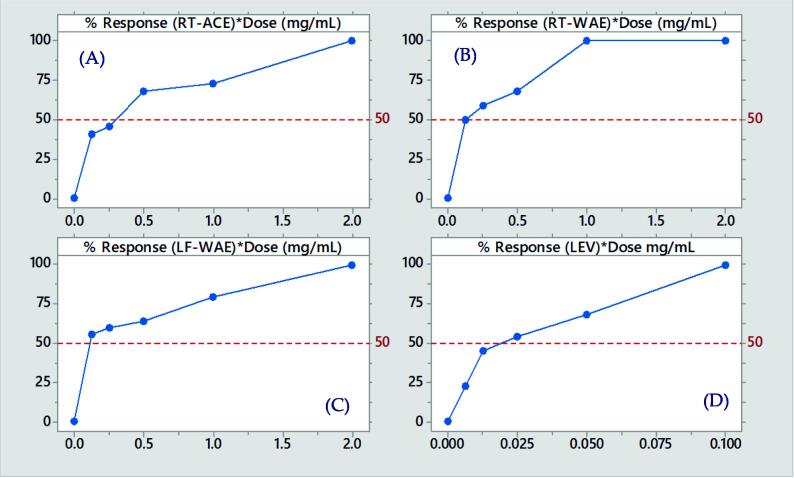
The dose–response curve of the most active extracts of *R. crispus* and levamisole on *C. elegans* at 72 h. The percentage response (lethality) is plotted against dose (mg/mL). (A) Response to acetone extract of root (RT-ACE) (B) Water extract of root (RT-WAE) (C) Water extract of leaf (LF-WAE) (D) Positive control, Levamisole (LEV).

**Fig. 2:**
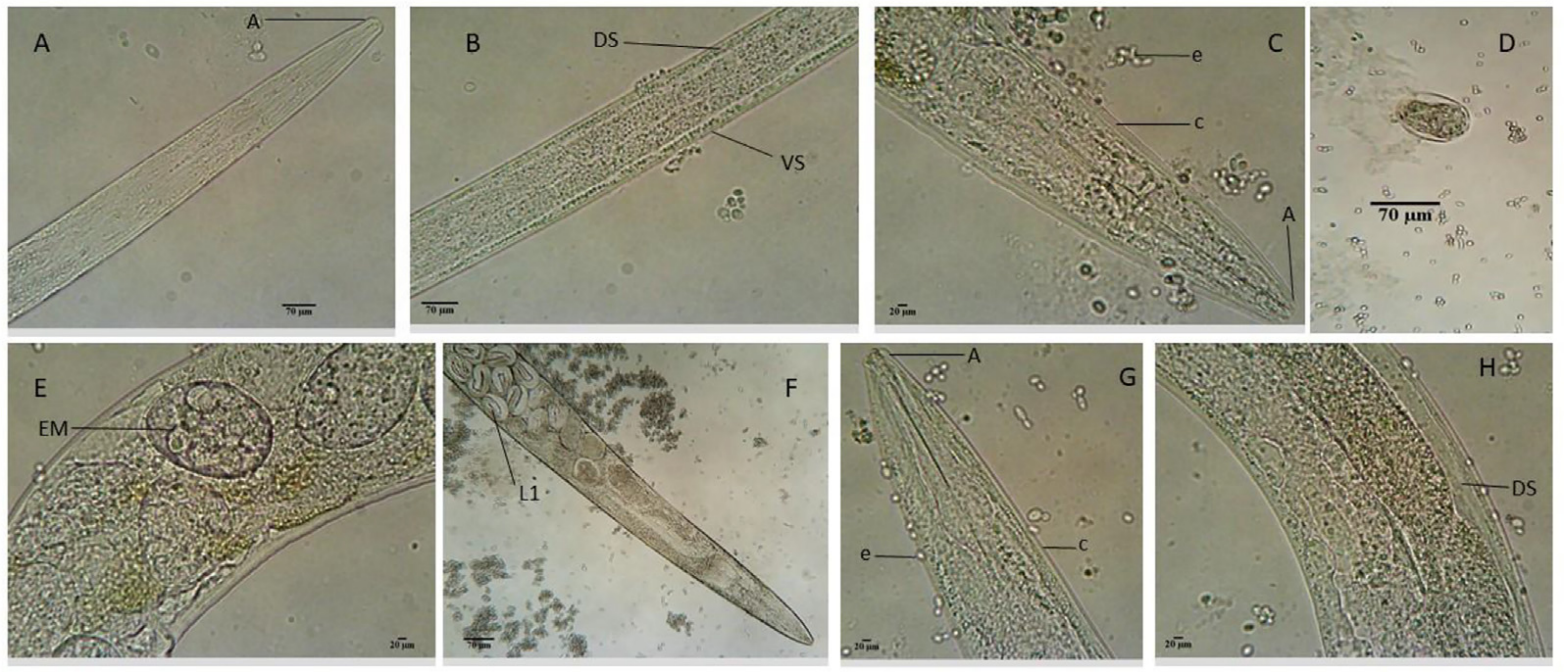
The morphology of treated *C. elegans* with 2 mg/mL extracts of *R. crispus* for 72 h. A- Untreated adult nematode (control) showing the anterior (A). B- shows the ventral side (VS) and dorsal side (DS) of the untreated nematode. C- Adult worm treated with RT-MEE. D - Egg treated *in vitro* with RT-MEE, shows a disoriented mass of aplasia formation of the L1 larva. E - Adults treated with RT-MEE show the development of *endotokia matricida* (EM) in some adults. F - Adults treated with RT-ETE, show developed *endotokia matricida* (EM) into an L1 larva (L1) in some of the adults. G – adult nematode treated with RT-ACE with cuticle (c) and eggs (e) around it. H – Adult treated with RT-ACE.

**Fig. 3:**
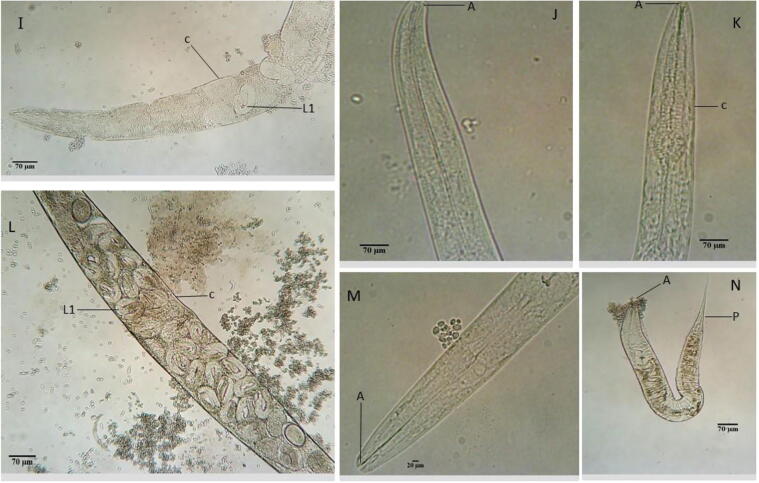
The Morphology of treated *C. elegans* for 72 h with 2 mg/mL *R. crispus* extracts and 0.1 mg/mL levamisole using a light microscope. I – Nematode treated with RT-WAE, showing developed endotokia matricida (EM), L1 larva (L1) and broken cuticle (c). J – Adult treated with LF-MEE, shows an intact body and the anterior (A). K - Adult treated with 0.1 mg/mL levamisole. L – Adult treated with LF-WAE, showing young nematode and thin cuticle after death (c) and developing L1 larva (l). M- Adult worm treated with LF-ACE, showing anterior (A). N - Nematode treated with LF-ETE, showing anterior (A) and posterior (P).

**Table 1:** The LC_50_ (mg/mL) and the confidence limits of the lethal concentration of extracts of the root and leaf of *R. crispus* against *C. elegans* for an exposure period of 72 h.

Plant Extracts	LC_50_ (mg/mL)
RT-MEE	3.27 (2.13–5.20)
RT-ETE	1.33 (1.02–1.85)
RT-ACE	0.29 (0.55–1.40)
RT-WAE	0.13 (−0.15–0.90)
LF-MEE	2.14 (1.41–7.51)
LF-ETE	2.67 (1.81–6.65)
LF-ACE	3.43 (2.32–7.12)
LF-WAE	0.11 (−0.52–0.65)
LEV	0.018

RT-MEE: methanol extract of the root; RT-ETE: ethanol extract of the root; RT-ACE: acetone extract of the root; RT-WAE: water extract of the root; LF-MEE; methanol extract of the leaf; LF-ETE: ethanol extract of the leaf; LF-ACE: acetone extract of the leaf; LF-WAE: water extract of the leaf.

### 3.2 Micromorphology analysis and effect of *R. crispus* on *C. elegans*

**Fig. 4:**
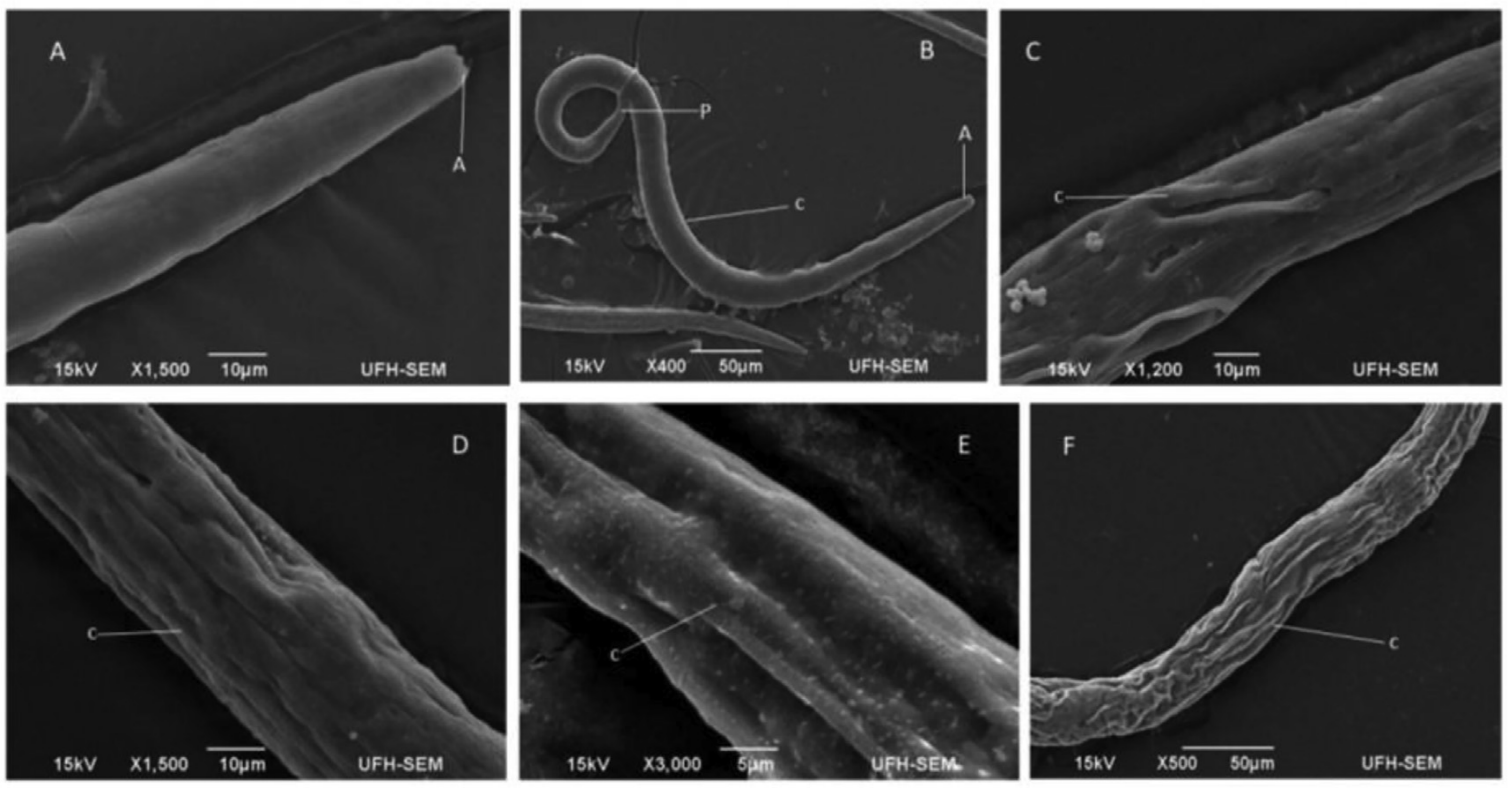
The morphology of treated *C. elegans* for 72 h with 2 mg/mL extracts of *R. crispus* using scanning electron microscopy (SEM). A- Untreated adult nematode (control) showing the anterior (A) with the oral opening. B - Untreated nematode showing the entire body with the anterior (A), the posterior (P) and intact cuticle (c). C - Adult nematode treated with RT-MEE, shows depression in the cuticle (c). D – Worm treated with RT-ETE, displays rough and reduced body. E – Adult nematode treated with RT-ACE shows reduced body and white patches on the cuticle (c). F - Nematode treated with 0.1 mg/mL levamisole shows extreme reduced body and rough cuticle.

**Fig. 5:**
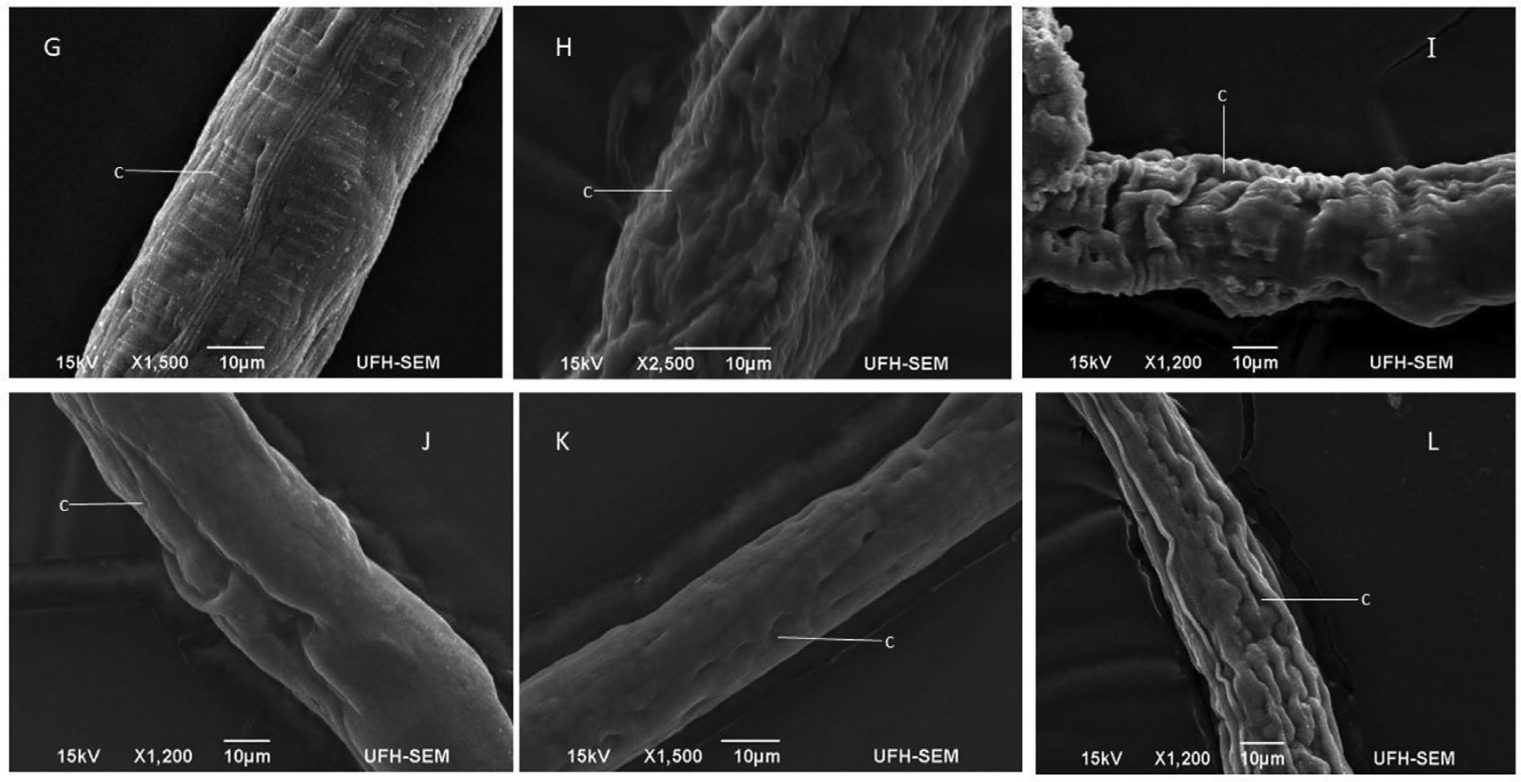
The morphology of treated *C. elegans* for 72 h with 2 mg/mL extracts of *R. crispus* and 0.1 mg/mL levamisole was examined using scanning electron microscopy (SEM). G - Adult worm treated with RT-WAE, shows stripes on the cuticle. H - Nematode treated with LF-MEE, shows severe damage to the cuticle (c). I – Adult worm treated with LF-ETE, shows a reduced body and wrinkled cuticle (c). J – Worms treated with LF-ACE show a smooth cuticle but slight body depression. K - Adult nematode treated with LF-WAE shows hollows in the cuticle (c) which might be perforated. L – Nematode treated with 0.1 mg/mL levamisole showed a reduced body and rough cuticle.

The effect of *R. crispus* on *C. elegans* was examined with SEM and an optical microscope. Fig. 2, Fig. 3, Fig. 4, Fig. 5 represents the average response of nematode per treatment. A light microscope was used to examine the adult *C. elegans* body wall, which is composed of an extracellular cuticle and a muscular layer that separates the interior from the environment. The internal organs were visible with a light microscope due to the transparency of the nematode; the pharynx, intestine, spermatheca, the formation of eggs, dorsal and ventral sides could be seen. In light microscopy, treated samples showed slight modification in the internal organs of the adult worms (Fig. 2, Fig. 3). Treatment of the nematode with 2 mg/mL plant extracts and 0.1 mg/mL levamisole for 72 h induced *endotokia matricida* in some of the adult worms, as shown in Figs. 2E, 2F, 3I, and 3L. The light microscope does not reveal the extent of extracts impact on the cuticle and body wall of the nematodes, but there is an observed structural modification, particularly in the internal organs of some of the nematodes. Fig. 2E and 2H reveal slight yellowish endogenous pigments which could have been from the extracts. Deformity was observed in the egg of the worm as shown in Fig. 2D. In Fig. 3N, it was observed that some nematodes treated with LF-ETE seem to give up some substance from the anterior region. We are uncertain what this substance was, but it may have been caused by the stressor (LF-ETE).

Scanning electron microscopy (SEM) was used to screen both treated and untreated adult nematodes. SEM revealed modifications such as lesions on the surface (cuticle) of the treated nematodes, which could have occurred due to the treatments (extracts). It was confirmed, after nematode exposure to treatments for 72 h, the formation of a rough cuticle (Fig. 4D and 4F, Fig. 5J and 5L), damage to the cuticle leading to hollows (Fig. 4C and 4F, Fig. 5I, 5 J, 5 K and 5L), spotted cuticle (Fig. 4E) and stripes on the cuticle (Fig. 5G).

### 3.3 Non-targeted screening of chemical constituent of *R. crispus*

#### 3.3.1 Fourier transform infrared Spectrophotometer (FTIR)

**Fig. 6:**
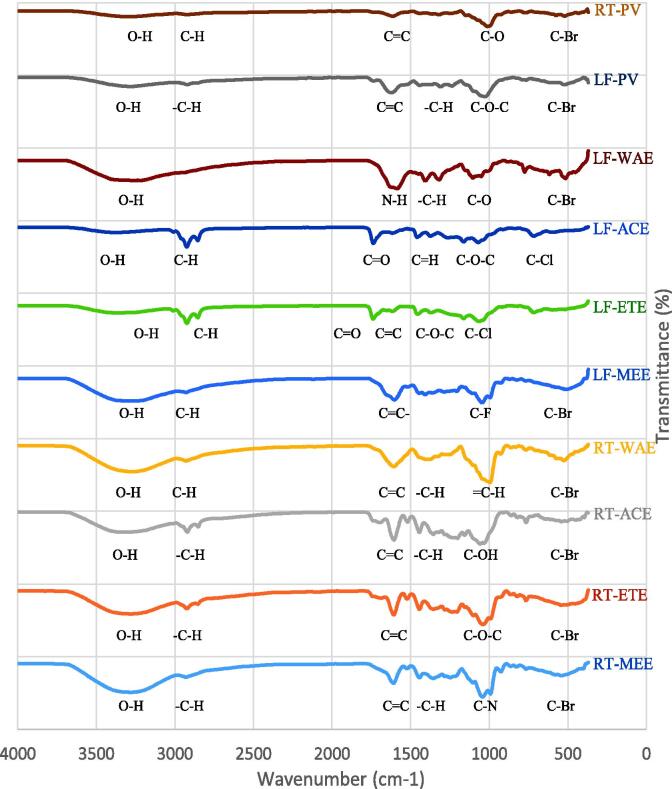
Overlaid FTIR spectra of *R. crispus* extracts and the pulverized samples. RT-MEE: Methanol extract of the root, RT-ETE: Ethanol extract of the root, RT-ACE: Acetone extract of the root, RT-WAE: Aqueous extract of the root, LF-MEE: Methanol extract of the leaf, LF-ETE: Ethanol extract of the leaf, LF-ACE: Acetone extract of the leaf, LF-WAE: Aqueous extract of the leaf, LF-PV: Pulverized leaf, RT-PV: Pulverized root.

The predominant bands in the absorption spectra of the samples of *R. crispus* were labelled in Fig. 6. There are quite many similarities between the functional groups of the spectra. In all the spectra; there was a broad absorption between 3600 and 3200 cm^−1^, which indicates the presence of a hydroxy group (O-H). All the bands have either or both the functional groups C-Br and C-Cl (Alkyl Halide), which are found in the fingerprint region of the band and they are known to be susceptible to nucleophilic substitution and/or elimination reactions. A C—N stretching vibration of aliphatic amines was observed in the region of 1047 cm^−1^ of the spectrum of RT-MEE, which is weak but aromatic amines, otherwise they are usually strong in the region of 1335–1250 cm^−1^ (Gao et al., 2013). A stretching vibration of a band of C—O—C (Alkyl-substituted ether) was observed in the spectra of RT-ETE, LF-ETE, LF-ACE and LF-PV, which could be mixed ethers. Ethers could be mixed if related to an alcohol group, when the hydrogen of the hydroxy group is replaced by an aliphatic (alkyl) or aromatic (aryl) molecular fragment (Coates, 2006). The alkene compound, carbon–carbon double bond (C=C), is present in all the spectra except LF-ACE and LF-WAE, which reveal C=O (ketone) and N—H (amide) respectively. The characteristic absorption frequencies for most parent organic hydrocarbon species are C—H stretching and bending vibrations, which were found in all the samples (Fig. 6). This justified the presence of complex hydrocarbons in the samples in the form of alkene and alkyne structures. The FTIR spectrum showed absorption bands representative of hydroxyl between 3433 and 3268 cm^−1^ for all samples, which is similar to the report of Liang et al. (2010), who isolated bioactive compounds from *Rumex nepalensis*. Likewise, the carbonyl group absorption bands (1730 cm^−1^ and 1710 cm^−1^) were found on LF-ACE and LF-ETE respectively, which is similar to the bands previously reported (Demirezer et al., 2001, Liang et al., 2010).

#### 3.3.2 Detection and identification of compounds in *R. crispus* using LC-MS

The annotated compounds in the extracts of *R. crispus* were characterized using LC-MS and the results are reported in Table 2. The protonated molecular ion peak at *m*/*z* = 290.2681 [M+H]^+^ in the ESI-MS revealed a calculated molecular formula of C_15_H_14_O_6_ annotated as (+)-Catechin. This base peak was found in all the extracts of *R. crispus*, indicating a high possibility of the presence of the tentative identified compound with the fragment ions; *m*/*z* 179, 203 and 245. The FTIR spectrum (Fig. 6) showed absorption bands between 3433 and 3268 cm^−1^ for all the samples, a strong indication of the hydroxyl group and a conjugated carbonyl group, which supports the possibility of (+)-catechin. Quercetin was tentatively characterized in the extracts of *R. crispus* as exhibiting precursor protonated molecular ion [M+H]^+^ at *m*/*z* 465.1008 with a molecular formula calculated for C_15_H_10_O_7_ and fragment ions at *m*/*z* 303.0486. The annotated cinnamtannin B-1 was calculated from the protonated molecular ion peak of *m*/*z* 865.1768 [M+H]^+^ and characterized as proanthocyanidin. The ion peak data of cinnamtannin B-1 was consistent with literature as cinnamtannin B1 was isolated from *Rumex acetosa* L. (Bicker et al., 2009).

**Table 2:** Identified compounds in the extracts of *R. crispus* using LC-MS.

						**Peak Areas**			
	Class of Compound	Annotated compound	Molecular formula	RT (Min)	Observed *m*/*z* ([M+H]+)	Methanol	Ethanol	Acetone	Water
Leaf extracts	Flavonoids	(+)-Catechin	C_15_H_14_O_6_	39.203	290.2681	8424942.042	1664393.879	4393690.14	1664393.879
		Quercetin	C_15_H_10_O_7_	20.774	303.0486	2370000.0	5709749.011	1303104.768	5709749.011

Root extracts	Steroidal alkaloid	Jervine	C_27_H_39_NO_3_	36.139	425.2997	3609346.942	–	–	–
	Flavonoids	(+)-Catechin	C_15_H_14_O_6_	39.309	290.2658	1657717.056	3264984.404	4858503.282	1757717.356
		Rotenone	C_23_H_22_O_6_	11.278	395.1259	–	4571454.455	–	–
	Proanthocyanidin	Cinnamtannin B-1	C_45_H_36_O_18_	14.000	865.1768	–	2434058.329	1180000.0	–

RT: Retention Time, - : compound not identified.

## 4 Discussion

The research leading to the discovery of a novel anthelmintic drug is expensive and has so far produced a fairly number of new compounds in the last decade (Csermely et al., 2013, Mackenzie and Geary, 2013). In this study, the anthelmintic potency of *R. crispus* extracts was appraised. The plant was selected based on its historical use by indigenous traditional healers to treat intestinal worm infections, inflammation and gastrointestinal disorders (Idris et al., 2017). The nematode *C. elegans,* was adopted as a surrogate model for parasitic nematodes as there are genomic homologs between free-living and parasitic worms (International Helminth Genomes Consortium, 2018, Mitreva et al., 2004) and is also an inexpensive system for drug screening. The effect of the treatment (plant extracts) on the nematode was observed using a light microscope and scanning electron microscopy (Fig. 2, Fig. 3, Fig. 4, Fig. 5). Phytochemicals are believed to correlate with the bioactivities of medicinal plants (Zhang et al., 2015). The phtochemicals of the extracts were analyzed using a Fourier Transform Infrared Spectrophotometer and Liquid Chromatography–High-Resolution Mass Spectrometry.

Interestingly, the most active extract of *R. crispus* against adult stage *C. elegans* was the water extract (leaf). After 72 h of exposure to the extracts (at 2 mg/mL), LF-WAE reduces the viability of the nematode by 83%, LC_50_: 0.11 mg/mL, which demonstrated great nematocidal activity compared to the reference compound (Fig. 1a). This study supports the research of Kumarasingha et al. (2014), who reported a strong potency of *Picria fel-terrae* (1 mg/mL) against adults *C. elegans*, compared to commercial drugs (Doramectin and Levamisole) used at 0.1 mg/ml. In addition to the lethality of nematodes, the extracts of RT-ETE, RT-ACE, RT-WAE, LT-WAE and levamisole induce the phenomenon in which the larval progeny hatch inside the adult *C. elegans*.

It was observed that most nematodes treated with RT-WAE, levamisole, and LF-WAE (Fig. 2: E and F, Fig. 3: I and L) presented the phenomenon known as *endotokia matricida*, which is probably caused by the ability of the extracts to induce starvation and oxidative stress in the nematodes (Sumaya et al., 2018, Trent et al., 1983). The phenomenon is a survival adaptation way that is common in species of the Rhabditidae family (Chen and Caswell-Chen, 2004) as a response to a stressor (s). The research reveals colouration in Fig. 2E and 2H, which might be inflammation caused by the extracts. The result of SEM used to further verify damages caused by these extracts on the surface of eggs and cuticles of the nematode revealed desquamation of various degrees, as shown in Fig. 5H and 5I. This was supported by the research done by Sant’anna et al. (2013), who also observed desquamation on the cuticle of nematodes treated with 25 µM albendazole using SEM.

The FTIR spectrum was used to characterize the functional groups of the compounds in the extracts of *R. crispus*. The FTIR spectra exhibit various characteristic strong vibration bands, fingerprint bands and several organic compounds at 3400–600 cm^−1^. They were phenols (C—O), aldehydes (C—H), ketones (C=O), alkene (C=C), organic acids (C=O), monocyclic aromatics (C—C, benzene), alkanes (C—C, C—H), alcohols (O—H), ethers (C—O), amines (N—H), amides (N—H), and alkyl halide (C—Br, C—Cl) (Gao et al., 2013, Ma et al., 2015). The compound’s characteristic peaks and functional groups are shown in Fig. 6. The characteristic stretching in the infrared absorbance in-between the wavenumber of 3000–2730 cm^−1^ indicated the existence of alkane (Gao et al., 2013). This was found in all the spectra except the aqueous extract of the leaf. The absorption bands at 1900–1660 cm^−1^ were related to C=O stretching, identified as ketone and aldehyde compounds. The bands are found in LF-ETE and LF-ACE. The absorbance bands at 1500–950 cm^−1^ were related to —C—H and C—O—H stretching bands and identified the groups of alkanes, phenols, esters or ethers. These bands are common to all the spectra in the extracts. The absorptions at 900–600 cm^−1^ bands were assigned to —C—H, C—Cl, and C—Br stretching for the group of alkyl halide and aromatic hydrocarbons, which were also found in all the spectra of the extracts.

To further investigate the bioactive compounds of *R. crispus* extracts, the separation was done by a chromatography phase coupled to mass spectroscopy where compounds were identified using a mass-to-charge ratio (*m*/*z*). The spectra fragmentation pattern was annotated, compared with the PubChem library and presented in Table 2. The main compounds identified are (+)-Catechin, Quercetin, Jervine, Rotenone and Cinnamtannin B-1 in *R. crispus*, constitute the complex mixture (extract) that could be responsible for the bioactive characteristics of *R. crispus*. Some compounds identified in this study matched those previously isolated from *Rumex aquaticus* (Orbán-Gyapai et al., 2017). However, catechin and quercetin were found in all of the extracts except the root extracts, where quercetin was not found. Quercetin has been reported with evidence of its promising effect as a potent anti-inflammatory and antioxidant compound which can be used against obesity (Nabavi et al., 2015). Jomova et al. (2017) reported quercetin has potent neuroprotective effects on patients with Alzheimer's disease and can protect against copper (II)-induced oxidative stress. Quercetin was confirmed to be the main compound in the extracts of *Momordica charantia* L. which shows high potency against Trichostrongylus, *Haemonchus contortus* and *Dictyocaulus viviparous* (Pereira et al., 2016). The presence of quercetin in the leaf extracts of *R. crispus* may justify the effectiveness against parasitic worms as used by traditional healers. Bicker et al. (2009), reported the isolation and identification of cinnamtannin B-1, (+)-catechin, and quercetin from another genus of Rumex (*R. acetosa*). These compounds were also identified in this study. The phytochemical, rotenone, is poisonous and yet it has been isolated from several plants, such as the root of *Derris elliptica* (Kang et al., 2016). It is confirmed to be an effective biopesticide phytochemical. Interestingly, a trace amount of rotenone was annotated from the calculated ESI-MS analysis of the ethanol extract of root (RT-ETE), an indication that the plant may not only be active against parasitic worms but could also be active against pests. The rotenone may also have contributed to the toxicity of *R. crispus* as reported (Idris et al., 2019b). This study suggested that the interaction of the complex mixture of the crude extract could be the source of the potency and the bioactivity of *R. crispus*. Hence, these complex compounds could have contributed to the medicinal properties of the plant.

## 5 Conclusion

A non-targeted approach was used to confirm the phytochemicals of *R. crispus* and the microscopy revealed the interaction and the potency of the plant extracts against the nematode. The study shows that the extracts of *R. crispus* are toxic to adults *C. elegans*, causing damage to both the internal as well as the body walls of the nematode. The bioactivity of *R. crispus* justifies its usage in ethnoveterinary medicine and by traditional healers for the treatment of gastrointestinal worm infections and stomach related problems. The plant may, therefore, serve as a good source of an anthelminthic novel drug.

## Author contributions

OAI conceived the study, carried out the research, analysed the data, interpreted the results and prepared the manuscript. OAW supervised the experiment at various stages, assisted in the writing and review of the manuscript. AJA provided the materials, equipment used, supervised and reviewed the manuscript with technical input. All authors have read and agreed to the publication of the manuscript.

## Funding

The authors received no funding from any external source.

## Declaration of Competing Interest

The authors declare that they have no known competing financial interests or personal relationships that could have appeared to influence the work reported in this paper.
